# Biological behavior of mesenchymal stem cells on poly-ε-caprolactone filaments and a strategy for tissue engineering of segments of the peripheral nerves

**DOI:** 10.1186/s13287-015-0121-2

**Published:** 2015-07-07

**Authors:** A. Carrier-Ruiz, F. Evaristo-Mendonça, R. Mendez-Otero, V. T. Ribeiro-Resende

**Affiliations:** Universidade Federal do Rio de Janeiro, Instituto de Biofísica Carlos Chagas Filho, Laboratório de Neuroquímica, Centro de Ciências da Saúde Bl. C, Cidade Universitária, 21949-900 Rio de Janeiro, RJ Brazil; Universidade Federal do Rio de Janeiro, Instituto de Biofísica Carlos Chagas Filho, Laboratório de Neurobiologia Celular e Molecular, Centro de Ciências da Saúde Bl. G Cidade Universitária, 21949-900 Rio de Janeiro, RJ Brazil; Universidade Federal do Rio de Janeiro, Núcleo Multidisciplinar de Pesquisa em Biologia - Numpex-Bio, Pólo de Xerém, Estrada de Xerém, N° 27, CEP: 25245-390 Duque de Caxias, RJ Brazil; Programa de Neurobiologia, Instituto de Biofísica Carlos Chagas Filho, UFRJ, Centro de Ciências da Saúde, Bloco C, Cidade Universitária, 21941-902 Rio de Janeiro, Brazil

## Abstract

**Introduction:**

Peripheral nerves may fail to regenerate across tube implants because these lack the microarchitecture of native nerves. Bone marrow mesenchymal stem cells (MSC) secrete soluble factors that improve the regeneration of the peripheral nerves. Also, microstructured poly-caprolactone (PCL) filaments are capable of inducing bands of Büngner and promote regeneration in the peripheral nervous system (PNS). We describe here the interaction between PCL filaments and MSC, aiming to optimize PNS tubular implants.

**Methods:**

MSC were plated on PCL filaments for 48 h and the adhesion profile, viability, proliferation and paracrine capacity were evaluated. Also, Schwann cells were plated on PCL filaments covered with MSC for 24 h to analyze the feasibility of the co-culture system. Moreover, E16 dorsal root ganglia were plated in contact with PCL filaments for 4 days to analyze neurite extension. Right sciatic nerves were exposed and a 10 mm nerve segment was removed. Distal and proximal stumps were reconnected inside a 14-mm polyethylene tube, leaving a gap of approximately 13 mm between the two stumps. Animals then received phosphate-buffered saline 1×, PCL filaments or PCL filaments previously incubated with MSC and, after 12 weeks, functional gait performance and histological analyses were made. Statistical analyses were made using Student’s unpaired *t*-test, one-way analysis of variance (ANOVA) or two-way ANOVA followed by Bonferroni post-test.

**Results:**

MSC were confined to lateral areas and ridges of PCL filaments, aligning along the longitudinal. MSC showed high viability (90 %), and their proliferation and secretion capabilities were not completely inhibited by the filaments. Schwann cells adhered to filaments plated with MSC, maintaining high viability (90 %). Neurites grew and extended over the surface of PCL filaments, reaching greater distances when over MSC-plated filaments. Axons showed more organized and myelinized fibers and reinnervated significantly more muscle fibers when they were previously implanted with MSC-covered PLC filaments. Moreover, animals with MSC-covered filaments showed increased functional recovery after 12 weeks.

**Conclusions:**

We provide evidence for the interaction among MSC, Schwann cells and PCL filaments, and we also demonstrate that this system can constitute a stable and permissive support for regeneration of segments of the peripheral nerves.

**Electronic supplementary material:**

The online version of this article (doi:10.1186/s13287-015-0121-2) contains supplementary material, which is available to authorized users.

## Introduction

The peripheral nervous system (PNS) consists of a complex network that extends throughout the body, maintaining contact with virtually all tissues and organs [1], and for this reason it is extremely vulnerable to injury. Annually, over one million people worldwide suffer from PNS lesions [2], and 120,000 patients a year in Brazil, the USA and Europe are subjected to peripheral nerve surgeries arising from domestic and traffic accidents, and tumor compression of axons, among other causes [3]. The PNS is capable of regenerating, even after a complete transection of axons [4]. This regenerative capacity is attributed to many factors, including Wallerian degeneration, immune response, Schwann cells (SC), extracellular matrix proteins, and the role of neurotrophins and gangliosides [5]. A critical step for the successful regeneration of peripheral axons is the formation of bands of Büngner, specialized structures formed by aligned and proliferative dedifferentiated SC that constitute a permissive pathway guide for the regenerative axons [4, 6]. However, when the lesion is too long, regeneration is not possible.

Synthetic conduits made from different materials have been widely tested, aiming to improve the regenerative capacity of the PNS [7, 8]. Among these, poly-caprolactone (PCL) is one of the best-studied biodegradable materials and has already been approved as a nerve scaffold by the US Food and Drug Administration [9, 10]. The stability against degradation, low melting point, and specific elastic characteristics of PCL are useful properties for the regeneration of extensive nerve tissue [11–13]. In addition to these favorable characteristics, PCL microstructured filaments were able to induce the formation of bands of Büngner [14].

In the last few years, the therapeutic role of mesenchymal stem cells (MSC), isolated from the bone marrow and other tissues [15, 16], has been studied [17] due to their tissue repair activity [18]. When transplanted in a mild peripheral nerve injury, MSC increased the survival of sensory and motor neurons, the proliferation of glial cells, the axonal outgrowth, and the number and thickness of myelinated fibers, and reduced muscle atrophy [19–22]. This beneficial role is commonly attributed to a paracrine effect of MSC due to the release of several factors by these cells. In addition, MSC can induce production, by the host tissue, of soluble molecules such as nerve growth factor (NGF), brain-derived neurotrophic factor (BDNF) and fibroblast growth factor-2 (FGF-2), which act directly on neurons and glial cells [19, 23–27], or vascular endothelial growth factor (VEGF) and hepatocyte growth factor (HGF), which promote angiogenesis and block the formation of scar tissue, respectively [28].

One way of further stimulating the regenerative cellular responses triggered by a nerve injury is to combine the use of conduits and cell therapies [2]. The first step when combining MSC with different materials, in a conduit or filament guide, is to assess the interaction of the cells with the material, since it is well known that chemical [29], physical [30, 31] and biological [32] modifications of the culture environment can modify the cellular behavior of MSC and, with that, their modulator effects [18]. In this study, we investigated the interaction of MSC with microstructured filaments of PCL as a possible alternative for the regeneration of peripheral nerve tissue in a model of severe sciatic nerve lesion. We observed that MSC interacted with PCL microstructured filaments and maintained cell properties including morphology, proliferation, CD90-positive phenotype, and trophic activity (expression and secretion of FGF-2). Moreover, the MSC/PCL filaments supported the adhesion of SC derived from regenerating nerve tissue and were able to support extensive axonal regeneration of peripheral neurons in vitro*.* In the in vivo experiments, we observed robust nerve tissue replacement, reinnervation and motor functional recovery 12 weeks after the implantation of MSC/PCL filaments in adult rats.

## Methods

### PCL filaments

**Fig. 1:**
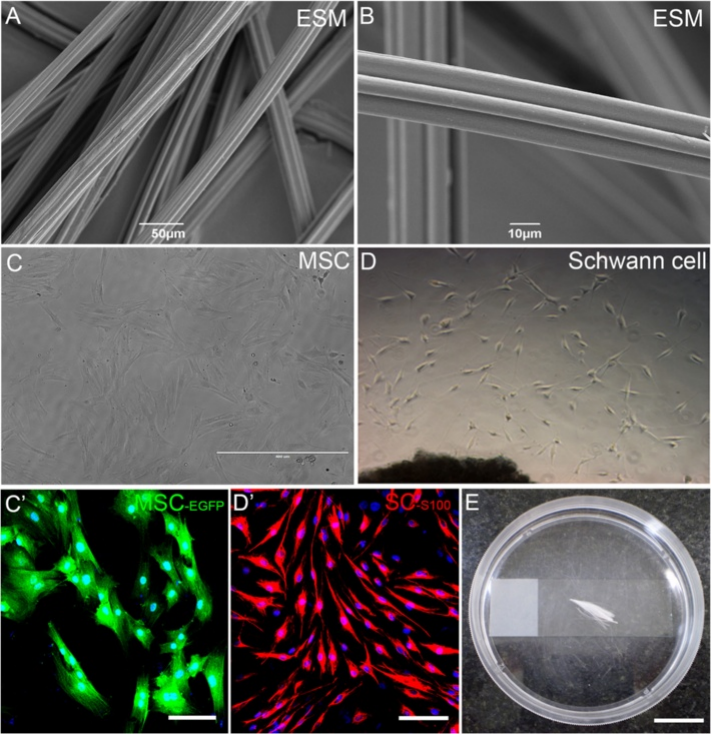
Setup for culture of PCL filaments with MSC or MSC with SC. **a**,**b** Scanning electron microscopy (ESM) photomicrographs of PCL filaments showing in low (**a**) and high (**b**) magnification the microstructured grooves formed by melting extrusion at 60 °C. **c** Phase-contrast photomicrograph and **c’** fluorescence photomicrograph in higher magnification of mesenchymal stem cells (MSC) in culture after three passages. In **c**’ MSC expressed enhanced green fluorescent protein (EGFP; green), and nuclei are labeled in blue (DAPI labeling). **d** Phase-contrast photomicrograph of Schwann cells (SC) derived from adult sciatic nerve cultured for 10 days. **d’** Fluorescence photomicrograph of SC immunostained for S-100 (red) and nuclei labeled with DAPI (blue). The vast majority of cells are positive for S-100. **e** Photograph illustrating the culture system used, with a bundle of PCL filaments treated with plasma-O_2_/poly-D-lysine/laminin and incubated with MSC or MSC plus SC for 48 h. Scale bars: **a** = 50 μm; **b** = 10 μm; **c**,**d** = 400 μm; **c**’,**d**’ = 100 μm; **e** = 2 cm

The PCL filaments used in this study were kindly donated by Prof. Dr. Burkhard Schlosshauer of The Natural and Medical Sciences Institute associated with the University of Tübingen, in collaboration with the Institute of Textile Technology and Process Engineering Denkendorf, Germany. Synthetic absorbable filaments were made from PCL with a molecular weight of 50,000 g/mol (Dow Tone, P767). Long microstructured filaments were formed by a technique of melting and extrusion in a spinneret at 205 °C, using a six-leaf nozzle with 24 capillaries. The yield volume was approximately 0.4 ml/min for each capillary, and the output speed was 1000 mm/min. In this state, the diameter of each capillary was 22 μm, with 66 μm of functional circumference. The bundles of filaments were washed with distilled water, wrapped around a microscope slide to facilitate handling, and the ends sealed. Small 2 cm segments (width of a microscope slide) containing hundreds of filaments sealed at the ends were sterilized in 70 % ethanol and dried [14]. Ultrastructural analysis was carried out by bonding the specimens in brackets covered with double-sided tape and imaging in an scanning electron microscope (Jeol JSM6390LV, JEOL, Peabody, MA, USA) after sputtering with a 20 nm thick gold layer (Fig. 1a,b). The functionalization of the filaments was performed in a three-step process. Initially, the material surface was hydrophilized using a glow discharge O_2_ plasma for three cycles of 75 s (PELCO easiGlow™, Pelco, Redding, CA, USA). Then, the filaments were coated with poly-D-lysine (50 μg/mL H_2_O; Sigma-Aldrich, São Paulo, SP, Brazil) and then coated with laminin (5 μg/ml; Life Technologies, Sao Paulo, Brazil).

### Animals

All experiments were performed following the National Institutes of Health Guidelines for the Care and Use of Laboratory Animals and approved by the Ethics Committee for the Use of Animals in Research from the Federal University of Rio de Janeiro (CEUA IBCCF protocol # 158). Lister hooded rats (3–5 months old) of both sexes were used, with a mean weight of 250 g (n = 40), bred at our institution’s rodent facility and housed with free access to food and water. In some experiments, we used Lewis rats (LEW-Tg (EGFP) F455.5/Rrrc), in which enhanced green fluorescent protein (EGFP) is expressed under the ubiquitin C promoter (n = 5); these rats were kindly donated by Dr. Alexandre Leite Rodrigues de Oliveira from the State University of Campinas, Brazil, and housed in our facilities.

### Mesenchymal stem cell culture

Male Lister hooded and EGFP Lewis rats were deeply anesthetized with an intraperitoneal injection of xylazine (5 mg/kg Rompum; Bayer, São Paulo, SP, Brazil) and ketamine chloride (50 mg/kg Vetaset; Fort Dodge Laboratories, New Jersey, NJ, USA) and killed by cervical dislocation. The tibias and femurs were removed and cleaned of muscle tissue, and the epiphyses were cut in a sterile environment. The bone marrow was flushed from the bones using 15 mL DMEM F-12 (Dulbecco’s modified Eagle medium) with 10 % fetal bovine serum (FBS; Life Technologies), penicillin and streptomycin (100 units/mL and 100 μg/mL, respectively; Sigma-Aldrich) and glutamine (1 mg/mL; Life Technologies), and the collected cells were gently dissociated with a Pasteur pipette. Cells (10^6^ cells/cm^2^) were added to culture dishes (96 × 21 mm; TPP, São Paulo, Brazil) and kept in an incubator with 5 % CO_2_ overnight at 37 °C. Nonadherent cells were removed, and adhered cells were supplemented with fresh culture medium three times a week. When the culture dishes reached 75–90 % confluence (Fig. 1c,c’), cells were trypsinized (0.25 % trypsin with 1 mM EDTA; Life Technologies), tested for viability with Trypan blue (0.2 %; Life Technologies) and expanded. MSC were used in experiments after 3 to 5 passages, with 75 to 90 % confluence [19].

### Schwann cell culture

Male Lister hooded and EGFP Lewis rats were deeply anesthetized as described above, then both sciatic nerves were crushed at the mid-thigh level for 15 s. After 24 h the animals were killed as described above and the nerves were carefully removed. The aim of this procedure was to activate SC at the distal stump after lesion, since the axons were disrupted. In a sterile environment, the epineurium was removed from the distal nerve stumps under low-magnification microscopy, and small pieces of nerve were removed with ophthalmic scissors. Nerve explants were placed in culture dishes (60 × 16 mm; TPP) with DMEM F-12, 20 % FBS, penicillin and streptomycin and glutamine, for 1 week to allow SC migration, with replacement of the culture medium three times a week. After this period, the culture medium was changed to 10 % FBS plus 10 ng/mL FGF-2 (Life Technologies) and 2 μM Forskolin (Sigma-Aldrich) to stimulate SC proliferation [33, 34]. After 1–2 weeks in this medium, the SC reached 75–90 % confluence and were trypsinized, tested for viability with Trypan blue, and expanded to poly-D-lysine- and laminin-coated culture dishes. SC were used in experiments after 1–5 passages, with 75–90 % confluence, and 80 % purity (Fig. 1d,d’).

### Cell culture on PCL filaments

A bundle of filaments (as above) was separated from the main body and deposited on a microscope slide, in a Petri dish (Fig. 1e). After PCL treatment with plasma-O_2_ and poly-D-lysine/laminin, three different MSC concentrations were seeded onto the filaments: 2 × 10^4^ cells/mL (n = 4), 8 × 10^5^ cells/mL (n = 4) and 8 × 10^5^ cells/mL (n = 4), in a volume of 100 μL of culture medium, to test the best concentration of MSC. MSC were kept in culture for 48 h, with medium replacement every 24 h. A concentration of 2 × 10^5^ cells/mL MSC was used in all the remaining experiments.

SC were also plated at a concentration of 2 × 10^5^ cells/mL for 48 h, when cultured alone, in a medium containing FGF-2 (10 ng/mL) and Forskolin (5 μg/mL). When in co-culture with MSC on PCL filaments, the SC were plated at a concentration of 10^5^ cells/mL, 24 h after the MSC plating, and were kept in culture for 24 h. In some experiments, before the co-culture, SC were incubated with the fluorescent marker CellTrace™ Far Red DDAO-SE (1:500; Life Technologies), for 40 min in an incubator with 5 % CO_2_ at 37 °C and then washed three times for 5 min each. In control experiments, to better evaluate the influence of the three-dimensional environment of the PCL filaments, we used a conventional culture of MSC, in which cells were plated on glass slides treated with poly-D-lysine and laminin and maintained in culture as long as the filaments were incubated with MSC.

After 48 h of co-culture of the filaments with the MSC, they were washed with phosphate-buffered saline (PBS) 1× and fixed with 4 % paraformaldehyde (4 % PFA in 0.1 M phosphate buffer, pH 7.4) for 20 min. Immunostaining was performed for the CD90 membrane antigen (Thy-1) present in MSC. The filaments were then separated by the edges, spread on a microscope slide, mounted with Vectashield (Vector, Burlingame, CA, USA) and analyzed by confocal microscopy (LSM 510 META, Zeiss, Jena, Germany). The number of MSC that adhered directly to the filaments or to other cells, as well as the total number of MSC, were quantified and normalized per millimeter of filament and compared among the different experimental groups.

To assess cell viability, the LIVE/DEAD® Viability/Cytotoxicity (Life Technologies) kit was used. After 48 h of cultivation with filaments, the MSC (n = 4) or MSC and SC co-culture (n = 4) were washed once with PBS 1× and incubated with green fluorescent calcein-AM (1:5000) and red fluorescent ethidium homodimer-1 (1:1000) in 1× PBS for 10 min in an incubator with 5 % CO_2_ at 37 °C. Then, the filaments were washed with PBS 1× and the cells analyzed by epifluorescence microscopy for live cells (EVOS®, Life Technologies). The ratio between the number of viable cells and total cell number was calculated and compared among the different experimental groups.

To investigate cell proliferation, after 48 h, MSC grown on glass coverslips (n = 8) and on PCL filaments (n = 8) were washed, fixed as described above, and immunostained for Ki-67, a marker of proliferating cells that is present in all phases of the cell cycle, except G0. After that, the filaments were separated by the edges, spread on a microscope slide, mounted with Vectashield (Vector) and analyzed by confocal microscopy (LSM 510 META, Zeiss). The number of Ki-67+ cells was counted and the ratio between this number and the total number of cells was calculated and compared among the different experimental groups.

Immunostaining for FGF-2 was performed after washing and fixation of the co-cultures of the filaments with MSC. After that, the filaments were separated by the edges, spread on a microscope slide, mounted with Vectashield (Vector), and analyzed by confocal microscopy. The levels of FGF-2 protein in supernatants from MSC cultures on coverslips or PCL filaments maintained in complete DMEM F-12 medium were determined by enzyme-linked immunosorbent assay (ELISA) using the Quantikine Human FGF-2 Immunoassay (R&D Systems, Mineapolis, MN, USA) in accordance with the manufacturer’s instructions.

### Dorsal root ganglia explant culture and neurite extension

Dorsal root ganglia (DRG) explants were obtained from E16 embryos. Pregnant rats were anesthetized and killed as described previously, and the embryos were immediately removed. DRG were dissected and then incubated in culture medium (as above) containing NGF (50 ng/mL; Life Technologies) for 1 h at 37 °C and 5 % CO_2_ before being plated onto the filaments (incubated or not with cells) in a 24-well plate (TPP) previously coated with poly-D-lysine and laminin. The system was maintained in culture for 4 days and the medium was replaced on the third day. After that, DRGs on glass coverslips (n = 11), on PCL filaments (n = 14), on PCL covered with MSC (n = 15), and on PCL covered with MSC plus SC (n = 16) were carefully washed and fixed as described below. Immunostaining for NF-200 was performed and the samples were mounted directly on a microscope slide with Vectashield and analyzed by confocal microscopy. The maximum distance of neurite extension from the center of the DRG was measured and quantified by Image-J 1.48v software (National Institutes of Health, Bethesda, MD, USA) and compared among the different experimental groups.

### Immunostaining procedures

After fixation with 4 % PFA, the samples were washed three times for 5 min each with PBS with Triton X-100 (0.1 % or 0.3 % for cytoplasm and nuclear antigens) or without (for membrane antigens) and incubated with 5 % Normal Goat Serum (NGS) in these solutions for 30 min at room temperature. Incubation with primary antibodies was performed for 2 h at room temperature, or overnight at 4 °C, followed by three washes with washing solution. Incubation with secondary antibodies and TO-PRO-3 nuclear dye (1:1000; Life Technologies) was performed in blocking solution for 2 h at room temperature and washed three times. Samples were mounted on microscope slides with Vectashield (Vector). Primary antibodies used: mouse monoclonal anti-CD90 (1:100; BD Biosciences, São Paulo, SP, Brazil); rabbit polyclonal anti-GFAP (1:400; Dako, Glastrup, Denmark); rabbit polyclonal anti-KI67 (1:400; Abcam, Cambridge, MA, USA); chicken anti-GFP (1:1000; Life Technologies); mouse monoclonal anti-FGF-2 (1:50, Millipore, Dallas, TX, USA); goat polyclonal anti-MBP (1:200; Santa Cruz Biotechnology, Jackson Laboratories, Jackson Imunno Research, West Grove, PA, USA); and rabbit polyclonal anti-NF-200 (1:200; Sigma-Aldrich). Secondary antibodies used: goat anti-mouse Cy3 (1:1000; Jackson Laboratories, Jackson Imunno Research, West Grove, PA, USA); goat anti-mouse Alexa Fluor 488 (1:1000; Life Technologies); goat anti-rabbit Alexa Fluor 555 (1:1000; Life Technologies); goat anti-chicken Alexa Fluor 488 (1:1000; Life Technologies); and donkey anti-goat Alexa Fluor 594 (1:800; Life Technologies). In these reactions, the PCL filaments were revealed by reflected light from the confocal microscope laser.

### Sciatic nerve lesion and PCL filament implantation

Total transection and connection of the sciatic nerve was performed under anesthesia as described above. The right sciatic nerves were exposed at the mid-thigh level, and an approximate 10 mm nerve segment was sectioned and removed. Distal and proximal stumps were reconnected and sutured inside a 14 mm polyethylene tube, leaving a gap of approximately 13 mm between the two stumps inside the tube. One group of rats received only 20 μL of PBS 1× injected into the tube (n = 3), and another group had the tube filled with PCL filaments coated with poly-D-lysine and laminin (n = 3). A third group was implanted with PCL filaments previously incubated with MSC (n = 3, 2 × 10^6^ cells/ml). PCL filaments were inserted into the tube using small tweezers. After the animals recovered from anesthesia, they were returned to the animal facility and kept with food and water ad libitum for 12 weeks.

### Behavioral tests

The functional performance of the animals was assessed using the CatWalk locomotion analysis system (Noldus Information Technology, Wageningen, Netherlands). Before surgery, the animals were trained daily for 2 weeks on a walkway of 1.5 m to conduct two uninterrupted consecutive runs along the walkway. Both during the training periods and after the surgery, the animals were motivated to cross to the other end of the walkway by the presence of a food pellet. The animals were starved for 4 h before each training period. At the end of the 2 weeks of training, all animals were making runs without interruptions, lasting between 2 and 4 s. After surgery, the animals were evaluated once a week, for 12 weeks; the day before each evaluation, animals were starved overnight [35]. Runs of each animal were recorded by the CatWalk apparatus, and the recorded data (at least two runs per animal) were analyzed with version 10.0 of the CatWalk program. The parameters analyzed were area and maximum intensity of the footprint, and swing speed of the paw. Values for all paws were recorded and the ratio of ipsilateral paw to contralateral paw for each parameter was calculated and compared among the different experimental groups.

### Histology procedures

Twelve weeks after surgery, rats were deeply anesthetized as described above and perfused with a 0.9 % saline solution for 10 min (10 mL/min; Masterflex, Cole-Palmer Instrument Co., East Bunker Court Vernon Hills, IL, USA) followed by 4 % PFA (20 min) and then 4 % PFA + 10 % sucrose solution (10 min). Ipsilateral sciatic nerves, DRGs (L5), lumbar spinal cord, and gastrocnemius muscles were removed and kept in a 30 % sucrose solution in phosphate buffer for 48 h. The nerves had the epineurium removed and were photographed under a magnifying glass (Leica S8AP0, Leica Microsystems, Nussloch GmbH, Wetzlar, Germany). After embedding in OCT mounting medium, longitudinal sections (20 μm) of the sciatic nerves and muscles were obtained using a cryostat (Leica CM 1850, Leica Microsystems, Nussloch GmbH) and mounted directly on gelatin + 0.05 % chromium alum pre-coated slides. Slides were stored at −20 °C for immunofluorescence procedures. For motor endplate analysis, we employed the immunofluorescence procedure for NF-200, followed by incubation with α-Bungarotoxin conjugated with Alexa 555 (1:200; Life Technologies).

For semi-thin sections the animals were anaesthetized using ketamine (100 mg/kg) and xylazine (15 mg/kg) and euthanized via transcardial perfusion using a fixative solution (4 % PFA and 2 % glutaraldehyde in 0.1 M phosphate buffer, pH 7.4). After perfusion, the sciatic nerve was collected. The middle segments of the nerves were immersed for 2 h in a solution of 2.5 % glutaraldehyde in 0.1 M phosphate buffer (pH 7.4), washed with 0.1 M cacodylate buffer (pH 7.4) and post-fixed for 90 min in 1 % osmium tetroxide containing 0.8 % potassium ferrocyanide and 5 mM calcium chloride in 0.1 M cacodylate buffer (pH 7.4). The segments were then washed in 0.1 M cacodylate buffer (pH 7.4) and stained with 1 % uranyl acetate overnight in the dark. The next day, the nerves were dehydrated using increasing concentrations of acetone (from 30 % to 100 %), infiltrated with Embed-812 resin (Electron Microscopy Sciences, Hatfield, PA, USA) and polymerized at 60 °C for 48 h. Semi-thin (500 nm) transverse sections were generated using an RMC MT-6000 ultramicrotome, Sorvall, Milton Freewater, OR, USA. The semi-thin sections were stained with 1 % Toluidine Blue and examined using a light microscope (Zeiss Axiovert).

### Image processing and statistical analysis

Images were processed using the Image-J 1.48v program (National Institutes of Health) and all data were analyzed using GraphPad Prism 5 (GraphPad Software, Inc., La Jolla, CA, USA). Statistical analyses were performed using Student’s unpaired *t*-test, one-way analysis of variance (ANOVA) followed by Bonferroni post-test for comparison of all pairs of columns, and two-way ANOVA followed by Bonferroni post-test comparing all pairs of columns. The confidence interval was 95 %, and all values are expressed as ± standard error of the mean (SEM).

## Results

### Cell adhesion and viability in the presence of PCL filaments

**Fig. 2:**
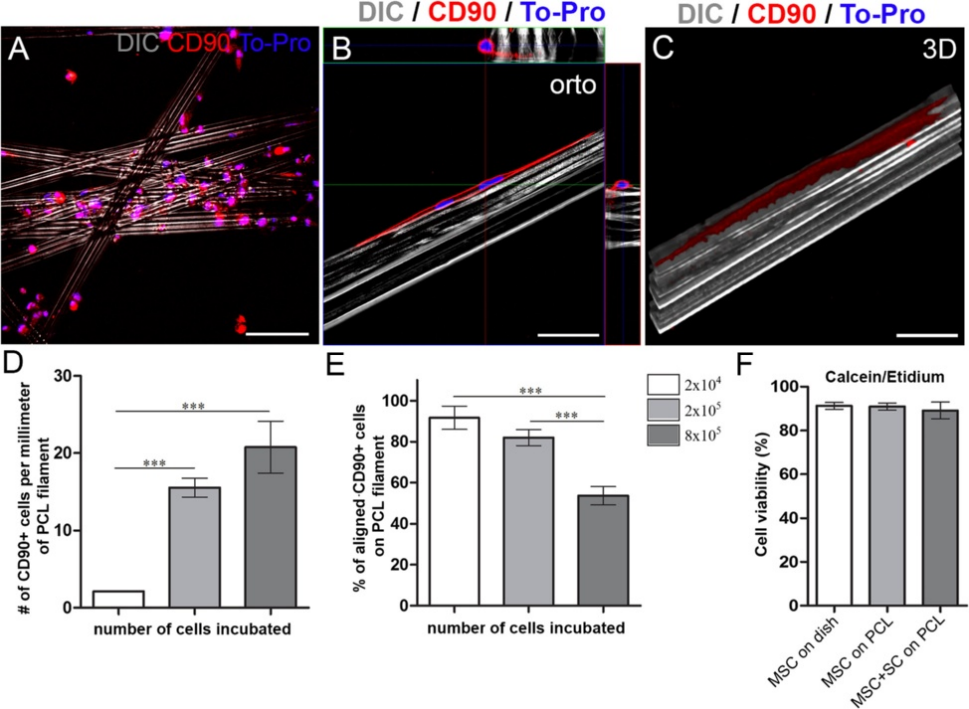
Cell adhesion and viability in the presence of poly-caprolactone (PCL) filaments. **a**, **b** Confocal microscopy optical sections of mesenchymal stem cells (MSC) incubated at medium density (2 × 10^6^ cells/mL) for 48 h on PCL filaments immunolabeled for CD90. **b** Orthogonal projections of Z-stack in high magnification, showing an individual PCL filament covered by two MCS. **c** Three-dimensional reconstruction by confocal microscopy of a single MSC (surface view) on an isolated PCL filament. **d** Quantitative analysis of the number of CD-90^+^ cells in contact with the PCL filaments and **e** the percentage of MSC aligned with the PCL filaments. **f** Percentage of viable cells in the different experimental conditions. Scale bars: **a** = 100 μm; **b**, **c** = 50 μm. ***p < 0.0001 by ANOVA. *SC* Schwann cells

To study the interaction between MSC and PCL filaments, a custom culture system was developed, consisting of a bundle of filaments deposited on a microscope slide in a Petri dish (Fig. 1e). Using this system, we first evaluated the interactions of MSC with the PCL filaments. After 48 h in culture, numerous CD90-positive cells were found in contact with the filaments (Fig. 2a), mainly over the ridges and longitudinally aligned, as seen in orthogonal planes with confocal microscopy (Fig. 2b). Furthermore, three-dimensional reconstruction of optical planes revealed that, although the cell nuclei were located on the ridges, the cell bodies and processes extended toward the grooves (Fig. 2c), thereby enlarging the area covered by the cells on the filaments.

We evaluated the best concentration of MSC by using three different cell densities and counting the number of cells in contact with the filaments (Fig. 2d). As the MSC plating number increased, so did the number of cells that were able to adhere to the material (Fig. 2d; *p* < 0.0001, no statistical difference between 2 × 10^5^ and 8 × 10^5^ cells, ANOVA). Evaluation of the cells longitudinally aligned with the filaments showed that the number of cells decreased as the plating number was increased, because with the highest density MSC adhered to each other instead of to the filaments (Fig. 2e; *p* < 0.0001, no statistical difference between 2 × 10^4^ and 2 × 10^5^ cells, ANOVA). Therefore, for the subsequent experiments, the plating concentration of 2 × 10^5^ cells (Fig. 2a) was chosen to maximize adhesion to and alignment with the PCL filaments.

The next step was to evaluate if the new culture environment would lead to alterations in MSC viability. Forty-eight hours after plating, the majority of MSC were stained with calcein-am (Additional file 1: Figure S1A), whereas only a few were stained with ethidium homodimer-1 (Additional file 1: Figure S1C), indicating high cellular viability. In a similar way, the viability of the system was also tested when SC were co-cultured with MSC. Again, the majority of the cells were stained with calcein-am (Additional file 1: Figure S1B), whereas only a few were stained with ethidium homodimer-1 (Additional file 1: Figure S1D). The MSC and SC could be distinguished from each other because SC were previously incubated with CellTrace (Additional file 1: Figure S1F). Merged images of MSC culture and MSC + SC co-culture are shown in Additional file 1: Figure S1G and Figure S1H. Also, there was no CellTrace staining in the MSC culture alone (Additional file 1: Figure S1E), as only the SC were previously incubated with CellTrace. As demonstrated by the quantitative analysis (Fig. 2f), the cells displayed high viability in the different experimental conditions.

### Proliferation and trophic activity of MSC in the presence of PCL filaments

**Fig. 3:**
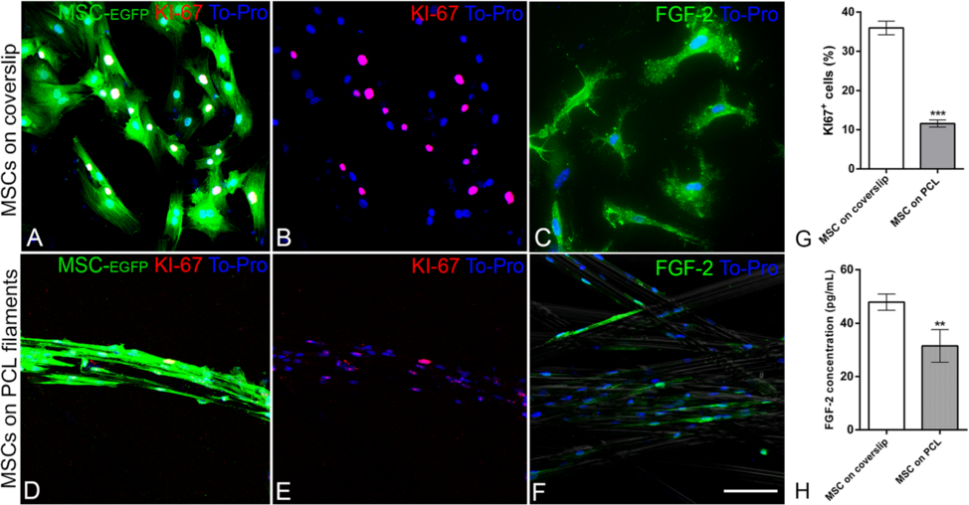
Cell proliferation and fibroblast growth factor-2 (FGF-2) expression in the presence of poly-caprolactone (PCL) filaments. **a**–**f** Confocal microscopy optical sections of enhanced green fluorescent protein (EGFP)-mesenchymal stem cells (MSC) cultured for 48 h on a coverslip (**a**–**c**) or on PCL filaments (**d**–**f**) immunolabeled for KI-67 (red) (**a**, **b**, **d**, **e**) or FGF-2 (**c**, **f**). Cell nuclei were stained with To-Pro (blue). **g** Quantitative analysis showing the percentage of KI-67+ MSC on a coverslip or PCL filaments. **h** Quantitative analysis of the FGF-2 concentration (pg/mL) detected by ELISA assay in the conditioned medium from MSC cultures on a coverslip or with PCL filaments. Scale bars: **a**–**f** = 100 μm. ***p < 0.0001, **p < 0.001, Mann-Whitney

To assess the cellular behavior of MSC plated on PLC filaments, the proliferation and expression of FGF-2, a trophic factor, was evaluated (Fig. 3). The number of cells that were positive for Ki-67 was used to quantify the proliferating cells. When MSC were cultured alone on coverslips, approximately 35 % of the cells were positive for Ki-67 (Fig. 3a,b,f). When the same cells were co-cultured with PCL filaments, the number of proliferating cells was reduced to approximately 10 % (Fig. 3d,e,g; *p* < 0.0001, Mann-Whitney).

The expression of FGF-2 by the MSC was investigated by immunostaining, and the release of FGF-2 to the culture medium was measured by ELISA. MSC expressed FGF-2 both alone and when in the presence of filaments (Fig. 3c,f). The amount of FGF-2 in the culture medium was reduced when MSC were co-cultured with the filaments (Fig. 3g; *p* < 0.001, Mann-Whitney).

### MSC/PCL biomaterial promotes neurite outgrowth of DRG neurons

**Fig. 4:**
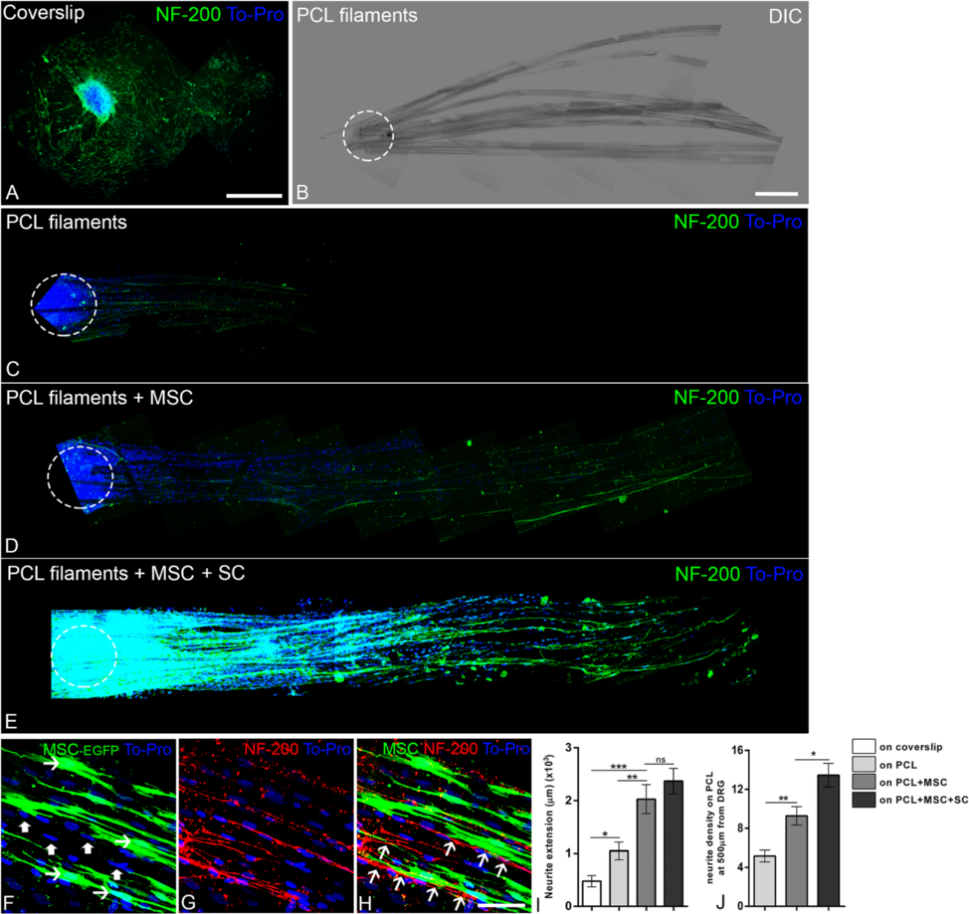
Neurite outgrowth from dorsal root ganglia (DRG) explants in the different culture conditions. **a**–**e** Confocal microscopy images of E16 rat DRG explants placed on a coverslip (**a**), on poly-caprolactone (PCL) filaments (**c**), on PCL filaments + msenshymal stem cells (MSC) (**d**), and on PCL filaments + MSC + Schwann cells (SC) (**e**) and cultured for 72 h. Neurites are identified by immunolabeling with NF-200 (green) and cell nuclei with To-Pro (blue). **b** Nomarski DIC image showing the organization of PCL filaments in these experiments. Dashed circles illustrate the area where DRGs were placed (**b**–**e**). **f**–**h** High-magnification confocal optical sections showing enhanced green fluorescent protein (EGFP) + MSCs (green) on PCL filaments (**f**) 72 h after incubation with DRG (thick arrows indicate MSC-EGFP and thin arrows indicate migrated non-MSC on PCL filaments). In (**g**), neurites were immunolabeled for NF-200 (red) and the merged image reveals neurites growing aligned in close association with MSC (**h**, *arrows*). **i,j** Histograms of quantitative analysis of maximum neurite extension (**i**) and neurite density on PCL filaments at 500 μm from DRG (**j**) after 72 h of incubation. Scale bars: **a** = 400 μm; **b**–**e** = 200 μm; **f**–**h** = 50 μm. *p < 0.01, **p < 0.001, ***p < 0.0001, ANOVA

DRGs were cultured over MSC/filaments to investigate a possible effect on neurite outgrowth. Neurites were immunostained for NF-200, and their maximum length was evaluated in the different culture conditions (Fig. 4). When DRGs were plated on coverslips, we observed a halo of NF-200-positive neurites extending radially from the explant. When DRGs were plated on filaments (Fig. 4b,c) we observed a preferential outgrowth of the neurites following the filaments (Fig. 4c). Most of the neurites were aligned with the filaments, and the maximum distance of neurite growth from the explant was twice that observed in DRGs plated on coverslips (Fig. 4a; *p* < 0.05, ANOVA). When the filaments were previously incubated with MSC (Fig. 4d) or with MSC + SC (Fig. 4e), the neurites extended over even longer distances compared with the DRG plated on coverslips (fourfold; *p* < 0.0001, ANOVA) or plated on PCL filaments alone (twofold; *p* < 0.001, ANOVA). There was no statistical difference between MSC and MSC + SC (Fig. 4i). We also counted the number of neurites at a distance of 500 μm from the explant (Fig. 4j). The largest number of neurites was observed in the presence of filaments plus MSC and SC (Fig. 4j). At higher magnification, we observed that DRG neurons extended neurites over migrated SC (GFP^−^, thick arrow) on the filaments, and were also in direct contact with the MSC (GFP^+^, thin arrow) on the filaments (Fig. 4f–h).

### The implantation of PCL/MSC biomaterial in adult rats improves nerve regeneration

**Fig. 5:**
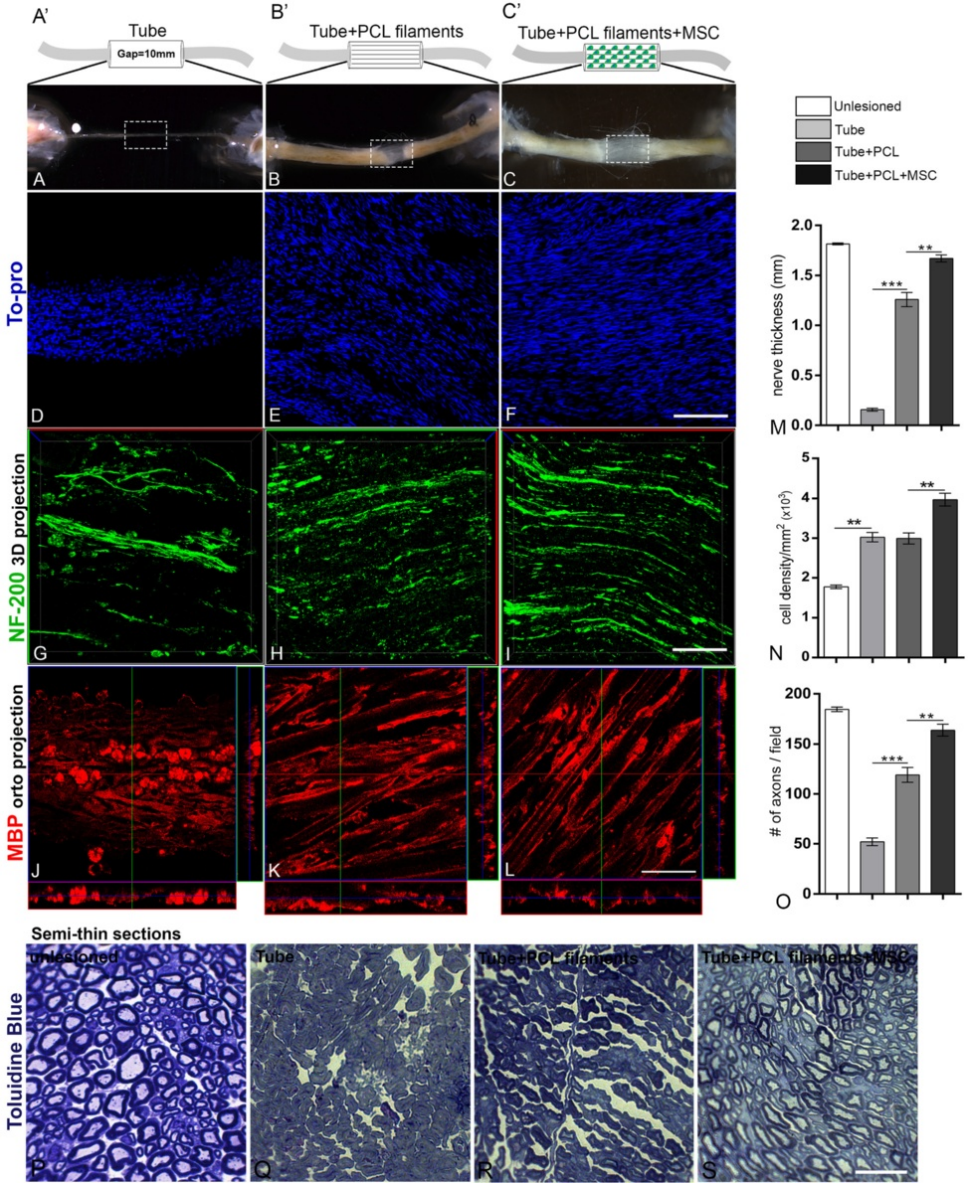
Regeneration of sciatic nerve after mesenchymal stem cell (MSC)/poly-caprolactone (PCL) implants. **a**’–**c**’ Schematic diagrams to illustrate the three in vivo experimental groups. **a**’ Tube with PBS; **b**’ tube with PCL filaments; **c**’ tube with PCL filaments plus MSC. **a**–**c** Low-magnification images of the sciatic nerves inside the tubes 12 weeks after surgery in the three conditions. Dashed squares indicate the areas imaged in **d**–**l. d**–**l** Longitudinal sections of the sciatic nerve tissue immunolabeled for NF-200 (**g**–**i**, green) and myelin basic protein (MBP) (**j**–**l**, red) with cell nuclei stained with To-Pro (**d**–**f**, blue). **m**–**o** Histograms of the quantitative analysis of the nerve thickness (**m**), cell density (**n**) and axonal density (**o**). **p**–**s** Photomicrographs by optical microscopy in high magnification of the semi-thin section of the regenerated nerves, 12 weeks after lesion and implantation stained with toluidine blue. Calibration bars: **a**–**c** = 5 mm; **d**–**l** = 100 μm; **p**–**s** = 20 μm. **p < 0.001, ***p < 0.0001, ANOVA; n = 6 animals for each experimental condition

For in vivo experiments, the experiment for implantation of PCL filaments into a polyethylene tube reconnecting both stumps (13 mm gap) after sciatic nerve transection was designed according to the illustrations in Fig. 5a’–c’.

Morphological macroscopic and quantitative analysis of the sciatic nerves in the three different experimental conditions showed important differences among the groups in the tissue present in the gap; regenerated tissue was present in the groups with filaments but only a thin regenerated tissue was observed in the PBS group (Fig. 5a–c,m). However, the group with filaments plus MSC had a thicker regeneration compared to the group with filaments (Fig. 5m; *p* < 0.001). Quantitative analysis of tissue sections of the three groups revealed a larger number of cells in the regenerated region in the group with the filaments covered with MSC, compared with the PBS group and filaments group (Fig. 5d–f,n). Due to the Wallerian degeneration, the cell density in all the experimental groups was raised compared to the unlesioned nerves. The presence of axons in this tissue was investigated by NF-200 staining, and more and better organized axons were present in the groups with filaments (Fig. 5g–i). Quantitative analysis demonstrates significantly increased axonal density in rats which received filaments covered with MSC compared to the filaments (0.4-fold; *p* < 0.01) and PBS group (1.6-fold; *p* < 0.001), respectively (Fig. 5o). Nerve tissue sections were also immunostained for S100 (not shown) and myelin basic protein (MBP), a marker of myelinating SC (Fig. 5 j–l). We observed MBP staining in all three groups, although in different patterns. In the PBS group, MBP expression was associated with cells at the periphery of the tissue (Fig. 5j), while in the filament groups the staining showed an elongated pattern, suggesting expression by myelinating SC associated with axons (Fig. 5k,l). We observed in the transversal semi-thin section of the regenerated nerves a progressive improvement in the tissue organization 12 weeks after lesion (Fig. 5p–s). The staining with toluidine blue revealed that the nerve histology of the animals receiving the filaments covered with MSC was similar to uninjured nerves, 12 weeks after lesion. Myelinating fibers can be easily observed in this group but not in the filaments and PBS groups (Fig. 5q–s). Taken together, these data demonstrate that bioengineered PCL filaments plus MSC promote consistent nerve tissue replacement with regular cell density, axonal regeneration, and myelinating SC replacement.

**Fig. 6:**
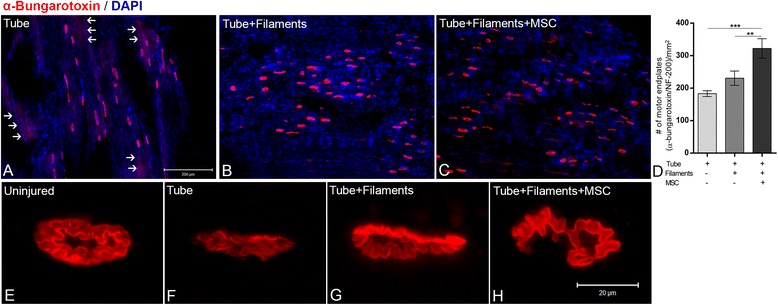
Motor endplate reinnervation. **a**–**c** Low-magnification images of longitudinal sections of the lateral gastrocnemius muscle labeled with α-bungarotoxin conjugated with Alexa 555 (red) 12 weeks after sciatic nerve lesion and implantation of hollow tube (**a**), tube + PCL filaments (**b**), or tube + PCL filaments covered with mesenchymal stem cells (MSC) (**c**). Cell nuclei were stained with DAPI (blue). *Arrows* in **a** indicate the red background of α-bungarotoxin which indicates the spread of the nicotinic receptor. **d** Histogram of quantitative analysis of the number of motor endplates (α-bungarotoxin/NF-200 positive units) per square millimeter, following the same experimental conditions as described above. **e**–**h** High-magnification images of isolated motor endplates (α-bungarotoxin) showing the morphology observed for uninjured (**e**), hollow tube (**f**), tube + PCL filaments (**g**), or tube + PCL filaments covered with MSC (**h**). Scale bars: **a**–**c** = 200 μm; **e**–**h** = 20 μm. **p < 0.001, ***p < 0.0001, ANOVA; n = 6 for each experimental condition

One important question is whether the regenerated axons are able to reinnervate the muscle. To answer this question, we obtained longitudinal sections of lateral gastrocnemius muscle, a main target of the sciatic nerve, and we double-labeled them with anti-NF-200 and with α-bungarotoxin (which binds strongly to the acetylcholine receptor) (Fig. 6a–c). The quantitative analysis demonstrated 63 % more motor endplates (α-bungarotoxin^+^/NF-200^+^) in the MSC group compared to the tube group, and 42 % more than in the filament group (Fig. 6d; *p* < 0.0001, ANOVA). At high magnification, we isolated individual motor endplates and compared the morphology of those from all experimental conditions with the muscle of unlesioned animals. Clearly, the MSC group showed similar morphology to the muscle of unlesioned rats (Fig. 6e–h).

### Functional effects of PCL/MSC biomaterial in adult rats with sciatic nerve lesion

**Fig. 7:**
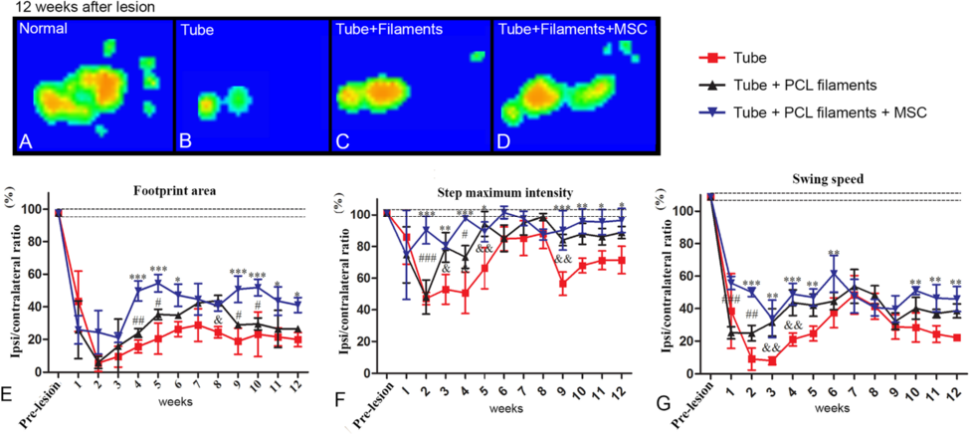
Functional benefits after the mesenchymal stem cell (MSC)/poly-caprolactone (PCL) biomaterial implants. **a**–**d** Footprint area and maximum step intensity (range from green to red) as observed in the CatWalk system, in the normal limb (**a**) and in the injured limb of the three groups of animals (**b**–**d**). **e**–**g** Histograms of correlation between the mean (ipsi/contralateral ratio) of the footprint area (**e**), maximum step intensity (**f**), and swing speed (**g**), measured every week for 12 weeks. *^,#,^&*p* < 0.05, **^,##,^&&*p* < 0.001, ***^,###^ *p* < 0.0001, ANOVA. *Tube versus MSC; &tube versus PCL; ^#^PCL versus MSC

The functional tests were performed 12 weeks after the surgery in the three groups. The footprint area and maximum step intensity in the affected hind limb were compared with the contralateral side and with the basal values before sciatic nerve lesion (Fig. 7a–f). The footprint area decreased to about 10–20 % of the pre-lesion values in all the groups 1 week after the surgeries (Fig. 7e). In all groups, recovery started 3 weeks after surgery, but improvement was significantly faster in the group that had received filaments plus MSC (*p* < 0.0001, ANOVA). The step maximum intensity also showed faster and better improvement in the groups with filaments, compared to the PBS group (Fig. 7f). The swing speed showed a similar pattern, with better performance in the filament + MSC group (Fig. 7g). Taken together, this set of experiments correlates the improvement in motor functional recovery with increased axonal regeneration, myelination, and innervated motor endplates in the animals that received the biomaterial containing filaments covered by MSCs.

## Discussion

The intrinsic regenerative capacity of the PNS is insufficient to completely restore its functions after extensive lesions [5]. In these cases, nerve transplants are performed in clinical practice, although this procedure involves some negative aspects [2]. To overcome these problems, new techniques of nerve tissue engineering have been explored, including the use of several types of conduits aiming to obtain equal or superior results compared with autologous transplantation [3, 8].

In our study, we used PCL microstructured filaments constructed to mimic the microarchitecture of a peripheral nerve, with six longitudinally oriented projections to induce the alignment of the SC and the formation of bands of Büngner in vitro. This composition forms permissive pathways for axonal regeneration and neurite growth over the biomaterial [14, 36].

In addition to structural modifications of the materials, the possibility of further improvement of axonal regeneration with cells was explored [17]. In many reports, bone marrow-derived MSC have been used to increase peripheral nerve regeneration by several mechanisms, which include an increase in SC proliferation and in neuronal survival, and regrowth and remyelination of motor and sensory axons [19–22].

Although some studies have demonstrated the interaction between PCL biomaterials and MSC, these studies typically use nanofibers [37], nanofiber scaffolds [38] or porous films [39]. The studies focus mainly on the morphology that MSC adopt in contact with the material, the maintenance of MSC multipotency [38], or commitment to a specific differentiation phenotype [37, 39]. This study is the first to investigate the interaction between MSC and filaments having structural features on a micrometric scale. The difference in scale enabled us to develop a method of culture using a bundle of filaments independent of any other culture surface, since the cells that adhere to the filaments do not adhere to the slide beneath it (Fig. 2). In studies that use PCL nanofibers, due to the scale and organization of these materials, the MSC, while adhering to the fibers, also form adhesion foci to the substrate under the fibers. Therefore, the micrometric structure used here could facilitate its implantation in vivo.

In the case of cell therapies combined with biomaterials, the number of cells used is highly important in order to observe beneficial effects [40]. The initial amount of MSC plated was enough to study its profile of adhesion to PCL filaments. However, the concentration used was insufficient to obtain a material with high cell density. Increasing the number of MSC plated proved effective for augmenting the cell density and consequently the covering of the filaments. However, when this cell number was raised, the MSC began to behave differently. In addition to aligning with the filaments, they began to adhere to each other (Fig. 2). This kind of adhesion increases the fragility of the attachment to the material, since a complex of cells is held by only a few cells that are directly attached to the filaments. Furthermore, the size of the cell cluster may act as a physical barrier to the regeneration of peripheral nerves, preventing the formation of optimized bands of Büngner by SC and reducing the available space for axonal extension.

The ability of MSC to adhere to the filaments does not necessarily mean that their physiology remains unchanged. Cell viability is an early indicator in this regard, since if the cells do not maintain high viability, they are less likely to produce beneficial effects. Cell viability was high when MSC were cultured on PCL filaments, contributing to the hypothesis that PCL filaments are not toxic to these cells (Fig. 2f). Even when cultured together with SC, a situation that would take place in vivo, which could result in competition between them for space and nutrients, both cell types maintained their high rates of viability. The coexistence of SC in apparent harmony with MSC in the established culture system can be explained by recent evidence that SC are present in certain niches of MSC [41], supporting the hematopoietic stem cell niche [42].

The analysis of Ki67 labeling (Fig. 3) showed that PCL filaments partially inhibit MSC proliferation when in direct contact. As the MSC are not in a traditional culture system for expansion, such as the surface of a culture plate, changes in their proliferation rate would be expected [37, 39]. It is possible that the morphology that MSC adopt when adhering to the filaments could be similar to that in their niche of origin. MSC proliferation also assumes similar rates to those observed in vivo, which indeed are low under physiological conditions [43]. A long-term study in monkeys demonstrated that MSC transplanted into a model PNS injury did not cause tumors to form [44]. If the filaments were to induce massive cell proliferation, the use of this in vivo system could be impaired due to the increased possibility of tumor formation in the transplanted area. Moreover, their continued proliferation rate of 10 % could contribute to the maintenance of cell renewal after transplant, by replacing a percentage of cells that die, thus maintaining their repair effect for a longer period of time.

The MSC paracrine ability was inferred by analyzing the production and secretion of FGF-2 (Fig. 3), since this trophic factor is highly important to the SC during the initial steps of Wallerian degeneration [26, 45]. The fact that these cells produce FGF-2 when cultured on PCL filaments, with only a small decrease in production, may indicate that the interaction with the biomaterial is not causing severe changes in their trophic activity. If these kinds of modifications were taking place, it could indicate an impairment of the paracrine effects of these cells for the PNS regeneration.

Some studies have shown that, when plated on longitudinally oriented nanofibers together with a cocktail of inducing factors, MSC are able to assume a commitment with neural phenotypes [45–47]. In the present study, even though the morphology and the rate of proliferation of MSC changed, due to the short culture time on the filaments (48 h) and the nonuse of inducing agents in the culture medium, it is unlikely that these cells are differentiating into a glial population. On the other hand, the decreased rate of proliferation, and the fact that they also express the CD90 membrane antigen and are able to produce FGF-2, could indicate a commitment to a repair phenotype [19].

Analysis of neurite outgrowth (Fig. 4) showed an increase in the distance and alignment of neurites when plated on filaments; these attributes can be extremely important to guide axonal regeneration in vivo. This alignment may prevent regenerating axons from growing toward the walls of the conduit. The fact that DRG neurites extend to greater distances when incubated on PCL filament covered with MSC shows that, even in a modified surface culture, MSC assist in the growth of neurites and could be even more beneficial for the regeneration of the PNS in vivo.

Detailed microscopic analysis showed that neurites grow over the SC which migrate from DRG, as occurs physiologically. Moreover, as the number of neurites increased and they made contacts with MSC, these cells can contribute to the permissive and correct pathway of regeneration. Also, the further increase in the number of neurites when MSC were incubated together with SC may indicate a synergistic interaction within the entire system.

Functional analysis by the CatWalk system (Fig. 7) showed that the utilization of PCL filaments was at no time detrimental to the animals in the parameters analyzed. Interestingly, the animals that received only the filaments showed intermediate values between the other two groups. In some cases they were closer to the values of the group with MSC, and in others, closer to the group in which only the lesion was performed. These observations plus the improved performance of animals with filaments and MSC clarify the importance of therapeutic effects with MSC in a model of critical injury of the sciatic nerve. However, it appears that the structural support offered by PCL filaments in events such as the induction of bands of Büngner by SC or axonal regeneration may be of great importance for the development of strategies for the regeneration of extensive nerve segments. The observation that PCL filaments covered by MSC significantly increased the number of healthy motor endplates suggests that this biomaterial was beneficial not only during acute periods of PNS regeneration, but also during the final steps of nerve regeneration (Fig. 6).

The temporal analysis showed that the eighth week after the injury is an important timeframe for regeneration in the lesion model used. Only animals that received PCL filaments with MSC did not show a regression in functional parameters at that time. One explanation for this may be that MSC, through their regenerative effect, induced a necessary prerequisite for effective regeneration of the PNS, such as acceleration or stabilization of axonal growth, thus preventing the functional regression observed in the other groups.

Twelve weeks after injury, the consequences of a critical injury to the sciatic nerve were evident (Fig. 5). Nerves of the “tube” group showed a frail regenerated tissue, with less cell density, consisting of few elements, such as fibroblasts, a few SC in the periphery, and secreted extracellular matrix molecules. Nerves of the “filaments” and “filaments + MSC” groups appeared to be more robust because of their increased cellularity and the presence of filaments, and showed greater SC migration and axonal regeneration. The more organized appearance of the tissue in the latter group may be a result of an increased maturation of the SC and stabilization of the axon by the reinnervation of the respective motor plate, induced by the MSC. Still, more detailed histological analysis will be needed in order to investigate the degree of remyelinization and the consequences to the neurons in the DRG and lumbar spinal cord, which project axons to the sciatic nerve, in each experimental group.

## Conclusion

In summary, this study contributes new information on the use of microstructured PCL filaments together with MSC for the regeneration of severe peripheral nerve injuries. Interaction with PCL filaments induces morphological and physiological changes in MSC, and positively modulates neurite outgrowth and alignment. This is the first time that these PCL filaments have been implanted in a model of critical lesion of the PNS, showing the feasibility of their use, the functional benefits and the histological organization of the regenerated nerve tissue, when combined with MSC.

## Additional file

Additional file 1: Figure S1. Cell viability in the presence of PCL filaments. A–H: Calcein/ethidium (live/dead) assay analyzed by fluorescence microscopy of MSCs co-cultured or not with Schwann cells (previously incubated with CellTrace) on the PCL filaments. A,B: Live cells revealed by calcein fluorescence of MSC (A) or MSC with Schwann cells (B). C,D: Dead cells revealed by ethidium fluorescence of MSC (C) or MSC with Schwann cells (D). CellTrace fluorescence of Schwann cells is observed in F (co-cultured with MSC) but not in E (only MSC). G,H: Merged images of calcein, ethidium and CellTrace, allowing the detection of MSC viability under both culture conditions. Scale bars: A–H = 400 μm.

## Abbreviations

ANOVA: Analysis of variance
BDNF: Brain-derived neurotrophic factor
CD90: Cluster of differentiation 90
DMEM: Dulbecco’s modified Eagle medium
DRG: Dorsal root ganglia
EDTA: Ethylenediaminetetraacetic acid
EGFP: Enhanced green fluorescent protein
ELISA: Enzyme-linked immunosorbent assay
FBS: Fetal bovine serum
FGF-2: Fibroblast growth factor-2
HGF: Hepatocyte growth factor
MBP: Myelin basic protein
MSC: Mesenchymal stem cells
NF-200: Neurofilament-200
NGF: Nerve growth factor
PBS: Phosphate buffered saline
PCL: Poly-caprolactone
PFA: Paraformaldehyde
PNS: Peripheral nervous system
SC: Schwann cells
VEGF: Vascular endothelial growth factor
