# Supplementary figures and images for: Biological behavior of mesenchymal stem cells on poly-ε-caprolactone filaments and a strategy for tissue engineering of segments of the peripheral nerves
Source: Stem Cell Res Ther. 2015 Jul 7;6(1):128. doi: 10.1186/s13287-015-0121-2 (PMC4522087; doi:10.1186/s13287-015-0121-2)

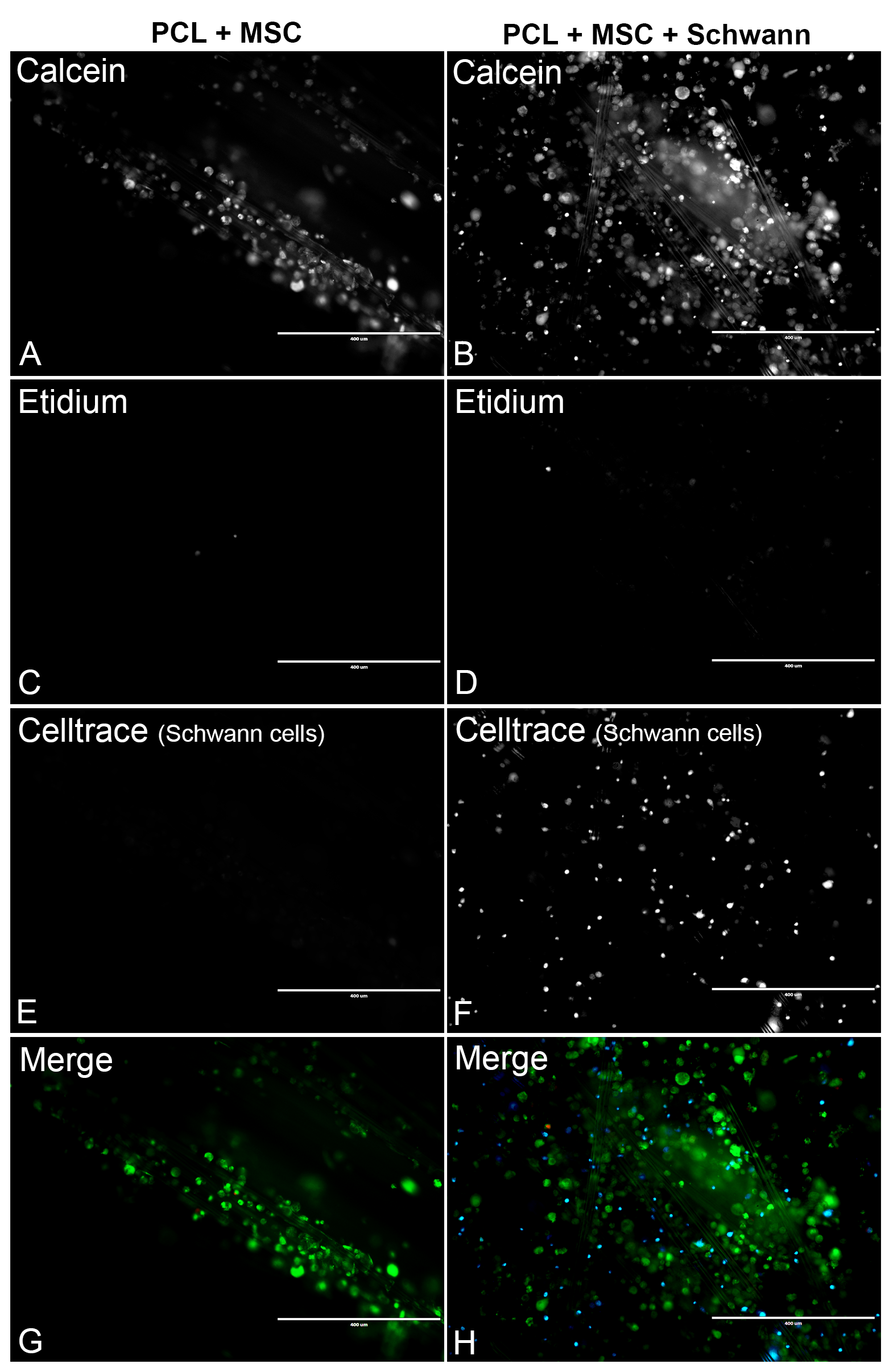

Supplement: Additional file 1: Figure S1. — Cell viability in the presence of PCL filaments. A–H: Calcein/ethidium (live/dead) assay analyzed by fluorescence microscopy of MSCs co-cultured or not with Schwann cells (previously incubated with CellTrace) on the PCL filaments. A,B: Live cells revealed by calcein fluorescence of MSC (A) or MSC with Schwann cells (B). C,D: Dead cells revealed by ethidium fluorescence of MSC (C) or MSC with Schwann cells (D). CellTrace fluorescence of Schwann cells is observed in F (co-cultured with MSC) but not in E (only MSC). G,H: Merged images of calcein, ethidium and CellTrace, allowing the detection of MSC viability under both culture conditions. Scale bars: A–H = 400 μm. [file 13287_2015_121_MOESM1_ESM.tif]
